# DepthDiff: Restoring Low-Depth Single-Cell RNA-Seq Signals via Diffusion Denoising

**DOI:** 10.3390/biology15151223

**Published:** 2026-07-23

**Authors:** Xiaojing Hou, Jinlei Sun, Yunqing Liu, Guoyong Wang

**Affiliations:** 1 School of Computer Science, Luoyang Institute of Science and Technology, Luoyang 471000, China; alisha@163.com (X.H.); liuyq@lit.edu.cn (Y.L.); 2 School of Information Engineering, Henan University of Science and Technology, Luoyang 471023, China; jlsun@lit.edu.cn

**Keywords:** single-cell RNA sequencing, expression enhancement, diffusion-based denoising, differential expression analysis, deep learning, low-depth scRNA-seq

## Abstract

**Background:** Low sequencing depth causes molecular capture loss and zero inflation in scRNA-seq data, reducing the reliability of downstream analyses. **Methods:** We propose DepthDiff, a depth-conditional expression enhancement model trained with diffusion-based denoising. Using low-depth expression profiles and sequencing depth ratio as conditions, DepthDiff learns a supervised residual mapping to paired high-depth references. During inference, it requires only a single-step forward prediction. Fixed-UMI downsampling was used to construct benchmarks across three public datasets. **Results:** DepthDiff outperformed supervised baselines, MAGIC, and unenhanced low-depth data in expression reconstruction and biological signal preservation. Ablation experiments showed that x0 prediction and cosine noise scheduling were important for stability, while full reverse diffusion sampling provided no additional benefit. Cross-dataset transfer and CITE-seq validation supported the generalizability and biological relevance of the recovered signals. **Conclusions:** DepthDiff is an efficient supervised framework for low-depth scRNA-seq expression enhancement, with gains mainly driven by diffusion-based denoising training rather than generative reverse sampling.

## Simple Summary

Low sequencing depth is a common bottleneck in single-cell RNA sequencing (scRNA-seq), leading to excess zero counts, distorted expression profiles, and reduced reliability of downstream analyses such as cell type identification and differential expression. Here, we propose DepthDiff, a diffusion-denoising-based deep learning model that learns to enhance low-depth expression profiles toward paired high-depth references. Unlike conventional diffusion models, DepthDiff performs only a single-step forward prediction during inference, avoiding costly iterative reverse sampling. Across fixed-UMI low-depth benchmarks from PBMC3K, PBMC68K, and human pancreas datasets, DepthDiff outperformed supervised baselines, MAGIC, and unenhanced low-depth data in expression reconstruction and biological signal preservation. DepthDiff provides an efficient and scalable framework for recovering reliable information from low-depth scRNA-seq data.

## 1. Introduction

Single-cell RNA sequencing (scRNA-seq) has become a core technology for dissecting cellular heterogeneity, cell-type composition, and transcriptional states [1,2]. However, the quality of transcriptomic data is essential for reliable biological inference, and sequencing depth directly determines the number of molecules observed in each cell [3]. In large-scale atlas construction, clinical sample screening, and cost-constrained experimental designs, researchers often obtain data with insufficient sequencing depth. Reduced UMI counts per cell, increased zero fractions, lower detection rates of lowly expressed genes, and altered cell-level library sizes jointly distort expression profiles, thereby affecting downstream tasks such as cell-type annotation, marker gene identification, differential expression analysis, and pathway analysis. Therefore, computational recovery of high-depth–like expression profiles from low-depth data has important scientific and practical value.

High-quality scRNA-seq reference data provide an ideal representation of expression patterns under a less degraded state, enabling a supervised enhancement strategy that learns a mapping from low-depth data to corresponding high-quality expression profiles. Existing expression imputation methods are mainly designed to address sparsity caused by low capture efficiency, such as graph-diffusion-based MAGIC [4], negative-binomial autoencoder-based DCA [5], and variational-inference-based scVI [6]. However, systematic benchmarks have shown that these methods can exhibit inconsistent performance across datasets and tasks, and some may introduce library-size-driven artifacts [7,8,9]. More importantly, many existing approaches rely on linear assumptions or predefined statistical distributions, which may limit their ability to model complex nonlinear dependencies among genes. In addition, their optimization objectives, including count likelihood, latent-variable modeling, or local smoothing, are not fully aligned with the specific goal of recovering high-depth–like expression profiles.

To address these limitations, we adopt the training paradigm of diffusion-based denoising. Denoising diffusion probabilistic models (DDPMs) [10,11] learn data distributions through progressive noise addition and reverse denoising, achieving success in image generation and noise inversion tasks [12,13,14,15]. Here, we analogize low sequencing depth to a degradation process that perturbs high-quality expression profiles and use multi-noise-level denoising as the training objective for expression enhancement. Importantly, DepthDiff uses the diffusion framework as a regularized denoising training objective rather than as a generative sampler. To meet the computational demands of large-scale omics data, DepthDiff performs only a single-step forward prediction during inference, directly learning an end-to-end mapping from low-depth to high-depth–like expression. Thus, DepthDiff should be understood as an efficient single-step, diffusion-denoising supervised enhancement framework rather than a full generative diffusion model. Its novelty lies in this task-specific integration and computational efficiency, rather than in proposing a new generative diffusion architecture.

In this study, we propose DepthDiff, a sequencing-depth-conditioned expression enhancement framework based on conditional diffusion-denoising training. Its key designs include using the residual between high- and low-depth profiles as the diffusion target, explicitly conditioning on the sequencing depth ratio d to enable a single model to handle multiple depths, and adopting x0 prediction with cosine noise scheduling to improve stability. We systematically evaluate DepthDiff across three datasets covering different biological contexts, compare it with supervised deep learning baselines and unsupervised imputation methods, and perform ablation experiments to clarify that the observed performance gains arise primarily from diffusion-based denoising training rather than generative reverse sampling.

## 2. Materials and Methods

### 2.1. Datasets and Preprocessing

This study used three public scRNA-seq datasets as high-depth reference data, covering diverse biological contexts from immune cells to solid tissue:

- PBMC3K: A peripheral blood mononuclear cell (PBMC) dataset from 10x Genomics (Pleasanton, CA, USA), containing approximately 2700 cells after quality control.
- PBMC68K: A large-scale PBMC dataset from 10x Genomics, containing approximately 67,000 cells after quality control, used to evaluate scalability.
- Pancreas: A human pancreatic inDrop dataset from Baron et al. (GEO: GSE84133) [16], with complex cell-type composition, used as a non-PBMC tissue validation dataset.

To ensure fair comparison across methods, all datasets were processed using the same preprocessing pipeline [17,18], which was kept identical for all compared methods. First, quality control was performed by filtering cells with extremely low or high numbers of detected genes or high mitochondrial gene proportions, and genes expressed in only very few cells were removed. Second, count matrices were normalized to 10,000 counts per cell (CP10k) and log1p-transformed. Third, feature selection was performed by selecting the top 2000 highly variable genes (HVGs) using only the training set, thereby avoiding information leakage from the test set. Cells were split into training, validation, and test sets at a ratio of 70%/15%/15%, with the random seed fixed at 2026.

### 2.2. Fixed-UMI Low-Depth Simulation

Because it is experimentally difficult to obtain strictly paired high-depth and low-depth profiles from the same biological samples, we adopted a standard strategy in the field: paired low-depth samples were generated from high-quality data through fixed-UMI downsampling. For each cell, the target UMI count was set to (d) times the median library size in the training-set HVG space, where (d) represents the sequencing depth ratio and takes values of 0.25, 0.50, and 0.75. Molecular-level sampling was then performed by binomial thinning of the original count matrix, using a per-gene retention probability that combines a gene-specific log-normal capture-efficiency factor with an expression-dependent dropout term that preferentially removes counts of lowly expressed genes. The simulated degradation is therefore non-uniform across genes rather than a purely uniform per-cell downsampling.

Compared with simple proportional downsampling, the fixed-UMI design makes cells at the same depth level have more comparable target molecular counts, thereby reducing confounding caused by library-size variation. All simulation parameters were estimated using only the training set to minimize data leakage. This procedure produced strictly paired low-depth and high-depth expression matrices at each depth level, which were used for supervised training and quantitative evaluation.

### 2.3. DepthDiff Model Architecture and Training

**Figure 1:**
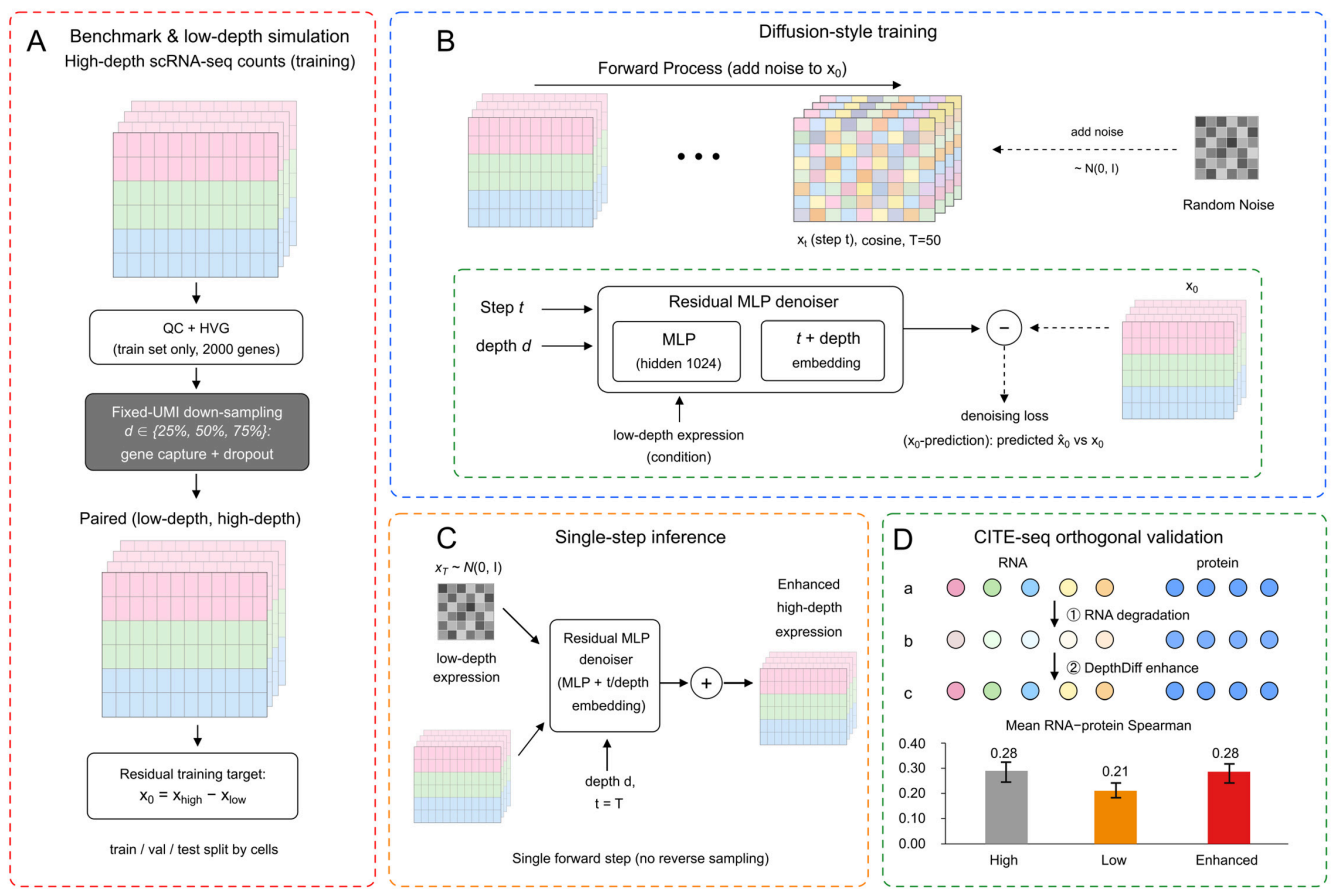
Conceptual framework of DepthDiff. (**A**) Data preprocessing and low-depth simulation. High-depth count matrices were processed by quality control and training-set HVG selection. Paired low-depth and high-depth samples were then generated using fixed-UMI downsampling at 25%, 50%, and 75% sequencing depths, incorporating gene capture and dropout effects. The model was trained to recover the residual difference between the low-depth and high-depth profiles. (**B**) Diffusion-based training. Gaussian noise was progressively added to the residual target according to a cosine noise schedule. A depth-conditioned residual MLP denoiser used the low-depth expression profile, noise level, and sequencing depth ratio as conditions to predict the clean residual signal. The denoising loss was computed between the predicted and true residual signals. (**C**) Single-step inference. Starting from noise, DepthDiff predicted the residual signal in a single forward pass conditioned on the low-depth expression profile and sequencing depth ratio. The enhanced high-depth-like expression profile was obtained by adding the predicted residual back to the low-depth input, without iterative reverse sampling. (**D**) CITE-seq orthogonal validation. RNA profiles were degraded and then enhanced by DepthDiff, and RNA–protein correlations were compared among high-depth, low-depth, and enhanced RNA profiles. Arrows indicate the direction of data flow through the workflow; the forward noise process in (**B**) and the RNA degradation and DepthDiff enhancement steps in (**D**) are labeled directly in the figure.

DepthDiff is an expression enhancement framework based on conditional diffusion-denoising training. The overall workflow is shown in Figure 1, including data preprocessing and low-depth simulation, diffusion-based training, single-step inference, and CITE-seq [19] orthogonal validation.

Forward Noise Process: The default diffusion target was defined as the residual between the high-depth and low-depth expression profiles:

(1) $$x_{0}=x^{high}-x^{low}$$

The forward noise process followed the standard DDPM formulation by progressively adding Gaussian noise to this residual target:

(2) $$x_{t}=\sqrt{{\bar{\alpha }}_{t}}x_{0}+\sqrt{1-{\bar{\alpha }}_{t}}\epsilon ,\epsilon ∼N\left(0,I\right)$$

where $\alpha_{t}=1-\beta_{t}$ and ${\bar{\alpha }}_{t}=\prod_{s=1}^{t}\alpha_{s}$. The noise variance $\beta_{t}$ followed the cosine noise schedule proposed by Nichol and Dhariwal [20], which makes the terminal state close to pure noise and improves the match between the training endpoint and the inference starting point. We used (*T* = 50) noise levels. This noise schedule was used only for multi-noise-level denoising during training; no multi-step reverse diffusion was performed during inference.

Denoising Network and Training Objective: The denoising network was implemented as a residual multilayer perceptron. Its inputs included the noised residual target $x_{t}$, the low-depth condition $x^{low}$, a time-step embedding, and a sequencing-depth embedding. Both the time step *t* and the sequencing depth ratio *d* were encoded using sinusoidal embeddings [21], allowing a single model to handle multiple sequencing depths. The network directly predicted the clean residual target ${\hat{x}}_{0}$ using $x_{0}$-prediction parameterization.

The training objective consisted of a depth-weighted denoising loss, supplemented by a sample-level correlation loss and a high-variance-gene variance preservation term applied to the reconstructed high-depth-like profile. Samples at 25% sequencing depth were assigned the highest weight to improve recovery performance under the most challenging low-depth condition.

Efficient Single-Step Inference: Unlike conventional DDPMs that require iterative reverse sampling, DepthDiff performed only a single-step forward prediction during inference. Specifically, $x_{t}$ was sampled from $N\left(0,I\right)$, corresponding to the terminal noise level (*t* = *T*). Conditioned on $x^{low}$ and sequencing depth ratio *d*, the model predicted ${\hat{x}}_{0}$ in one forward pass. The enhanced expression profile was then reconstructed as follows:

(3) $${\hat{x}}^{high}=clamp\left(x^{low}+{\hat{x}}_{0},0\right)$$

We also implemented full reverse diffusion chains, including DDPM ancestral sampling and DDIM sampling [22]. However, the ablation experiments in Section 3.6 showed that full reverse sampling provided no performance gain over single-step prediction and was substantially slower. Therefore, DepthDiff used diffusion as a multi-noise-level denoising training objective rather than as a generative reverse sampler.

The denoising network used a hidden dimension of 1024 and multiple residual blocks. The output dimension was equal to the number of selected genes.

### 2.4. Comparison Methods and Fair Benchmarking Protocol

We divided the comparison methods into three groups according to their training paradigms. Supervised paired enhancement methods, which used paired high-depth profiles as targets in the same manner as DepthDiff, included kNN, cVAE, and MLP. For kNN, the 15 nearest training cells were retrieved in the low-depth expression space, and the mean of their paired high-depth profiles was used as the prediction. The cVAE was implemented as a supervised conditional variational autoencoder [23], and the MLP was implemented as a direct supervised regression model. Unsupervised methods, which were trained only on low-depth counts and never used paired high-depth profiles, included MAGIC, a graph-diffusion-based smoothing imputation method implemented using the official magic-impute package. Reference data consisted of the unenhanced low-depth data, denoted as Raw.

To ensure a reliable comparison, all methods used the same preprocessing pipeline, the same training, validation, and test splits, and the same feature set. DepthDiff and the supervised neural-network baselines were trained with the same number of epochs (80 epochs), early stopping, and the AdamW optimizer [24], with a learning rate of $1\times 10^{-3}$ and a weight decay of $1\times 10^{-4}$. The unsupervised MAGIC baseline performed graph-diffusion smoothing on low-depth expression data in the same log1p(CP10k) space as the target, and its output was compared with the same high-depth reference. It should be noted that MAGIC only observes low-depth data and outputs dense smoothed expression profiles, which differs from the sparse high-depth-like reconstruction setting considered in this study. We nevertheless included MAGIC in the main comparison and report its performance transparently, with further interpretation provided in the Section 4.

### 2.5. Training Configuration and Evaluation Metrics

All analyses were implemented in Python (version 3.10) using PyTorch (version 2.1); the complete list of software dependencies and their versions is provided in the public code repository. All training was performed on a single NVIDIA RTX 4060 GPU (NVIDIA Corporation, Santa Clara, CA, USA) with fixed random seeds. Data processing, model training, and evaluation were organized as separately executable scripts to ensure reproducibility. Inference runtime (reported in Section 3.10) was measured on the same GPU using CUDA-synchronized timing averaged over repeated runs.

The evaluation metrics were divided into two categories. Technical metrics included root mean square error (RMSE; lower is better) and Pearson correlation (higher is better). Biological signal metrics included top-100 differentially expressed gene overlap (DEG overlap) calculated based on KMeans clustering with (*k* = 8), logFC Spearman correlation, marker gene Spearman correlation, and clustering consistency measured by ARI and NMI. All metrics were calculated on the held-out test set. Statistical significance was assessed using paired tests: paired *t*-tests were performed on per-cell Pearson correlations between DepthDiff and each baseline, and Cohen’s *d* was reported as the effect size.

### 2.6. Ethics Statement

This study used publicly available and de-identified datasets. The original studies obtained the relevant institutional review board approvals and informed consent from participants. The secondary analysis in this study complied with the data use policies of the corresponding databases.

## 3. Results

We systematically evaluated DepthDiff on three benchmark datasets. The comparison methods included three supervised paired enhancement methods (kNN, cVAE, and MLP), the classical unsupervised imputation method MAGIC, and unenhanced low-depth data, denoted as Raw.

### 3.1. DepthDiff Consistently Achieves the Best Overall Performance

Table 1 summarizes the technical metrics, and Table 2 summarizes the biological signal metrics across three datasets and three sequencing depths. Overall, single-step DepthDiff achieved the best Pearson correlation in all nine dataset-depth combinations, with a tie with Raw at 75% depth on PBMC3K. It also achieved the best DEG top-100 overlap in all nine combinations and the lowest RMSE in eight of nine combinations, with a tie with MLP at 25% depth on PBMC68K. These results indicate that DepthDiff outperformed all supervised baselines, MAGIC, and Raw in both expression reconstruction and biological signal preservation.

**Figure 2:**
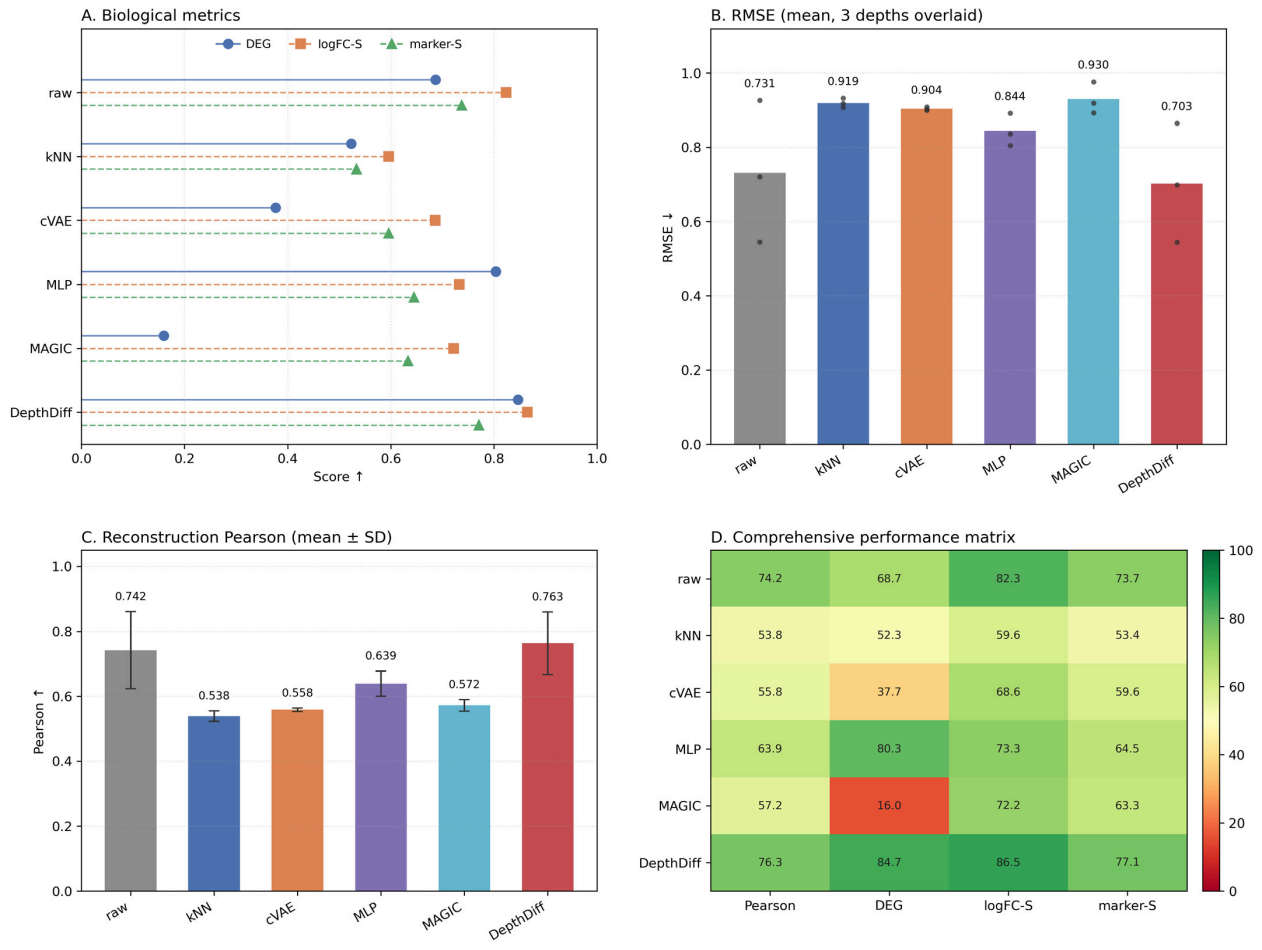
Comprehensive performance of compared methods on the PBMC3K dataset. Statistics are averaged across the three sequencing depths for this dataset. (**A**) Dot plot of biological signal metrics, showing mean values of top-100 DEG overlap, logFC Spearman correlation, and marker gene Spearman correlation for each method. (**B**) Bar plot of mean RMSE (lower = better), overlaid with individual data points for each of the three sequencing depths. (**C**) Mean ± standard deviation of reconstruction Pearson correlation (higher = better), with mean values labeled above each bar. (**D**) Composite performance heatmap showing mean values (scaled ×100) of four core metrics—Pearson correlation, DEG overlap, logFC Spearman correlation, and marker gene Spearman correlation—for each method; the color gradient ranges from red to green, indicating lower to higher performance. DepthDiff achieved the best performance across all four panels.

**Figure 3:**
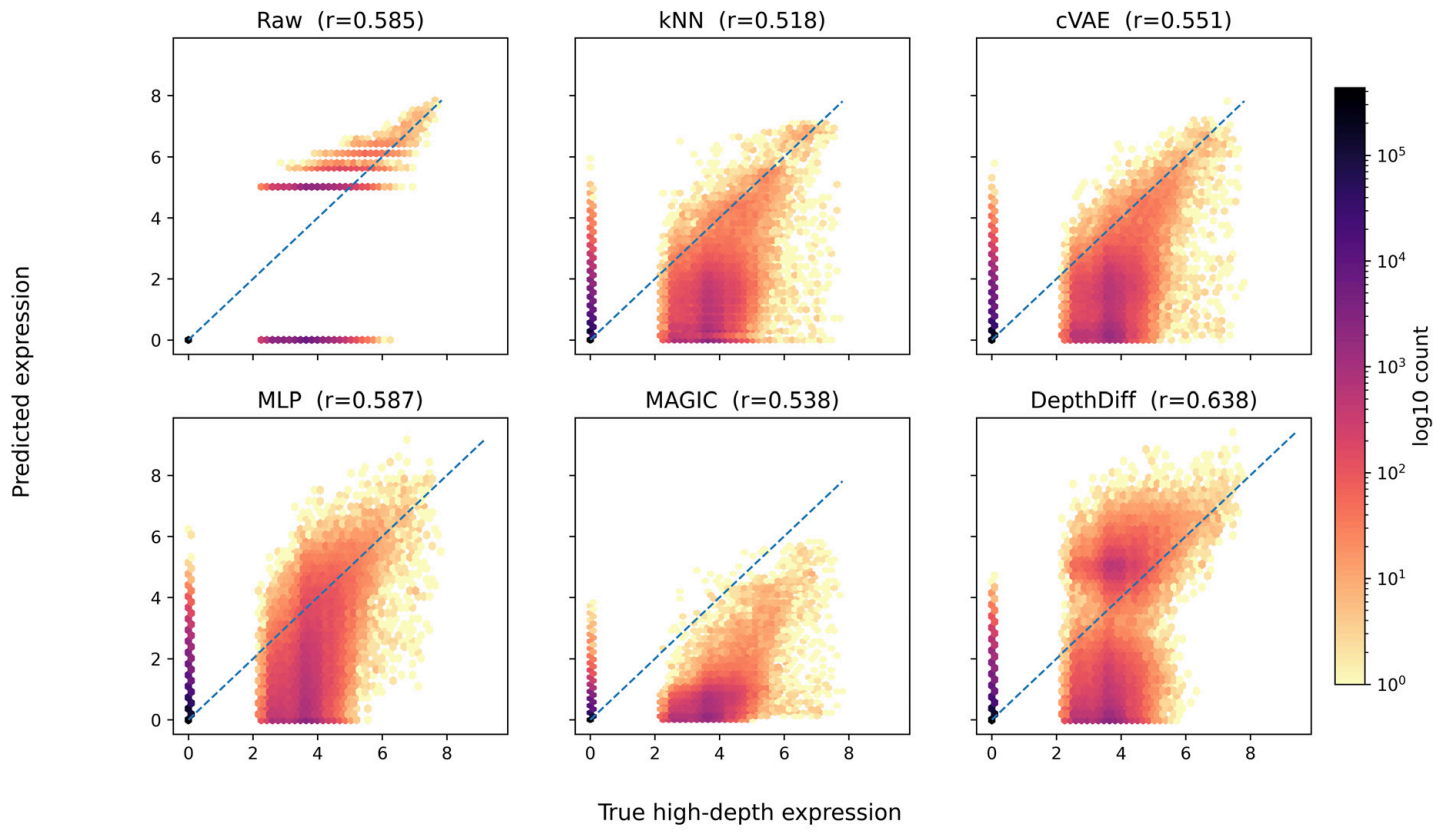
Two-dimensional density plots of predicted versus reference high-depth expression on PBMC3K at 25% sequencing depth. The six panels correspond to Raw, kNN, cVAE, MLP, MAGIC, and DepthDiff. The *x*-axis represents reference high-depth expression, and the *y*-axis represents predicted expression from each method. Color intensity indicates log-scaled count density, and the blue dashed line marks the diagonal. The global (flat) Pearson correlation for each method is shown in parentheses in the panel title. DepthDiff shows the closest alignment with the diagonal.

**Figure 4:**
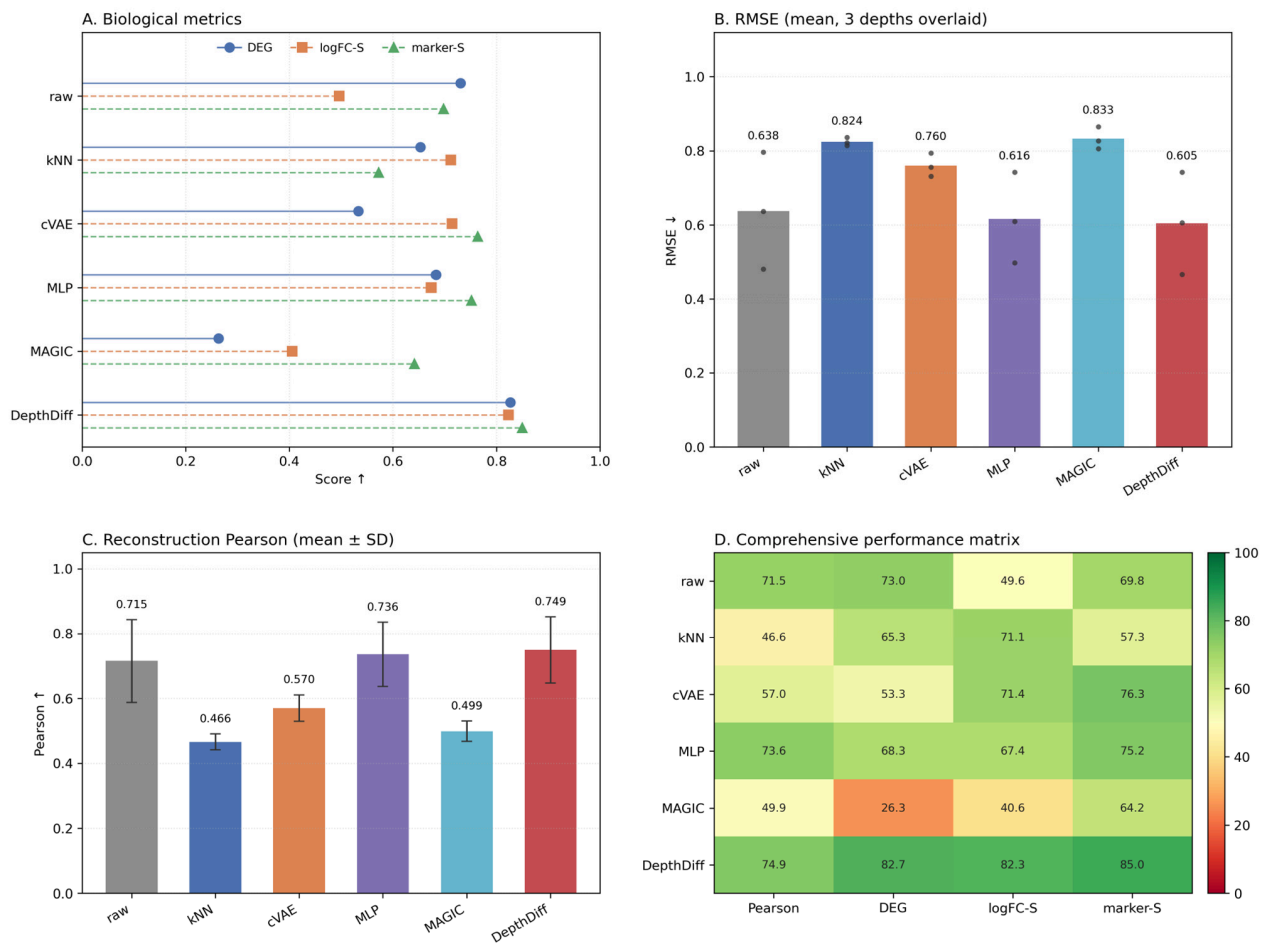
Comprehensive performance of compared methods on the PBMC68K dataset. Statistics are averaged across the three sequencing depths for this dataset. The four panels are defined as in Figure 2. In this larger-scale and more heterogeneous setting, DepthDiff maintained the best overall performance in biological signal metrics, RMSE, reconstruction Pearson correlation, and the composite performance heatmap.

**Figure 5:**
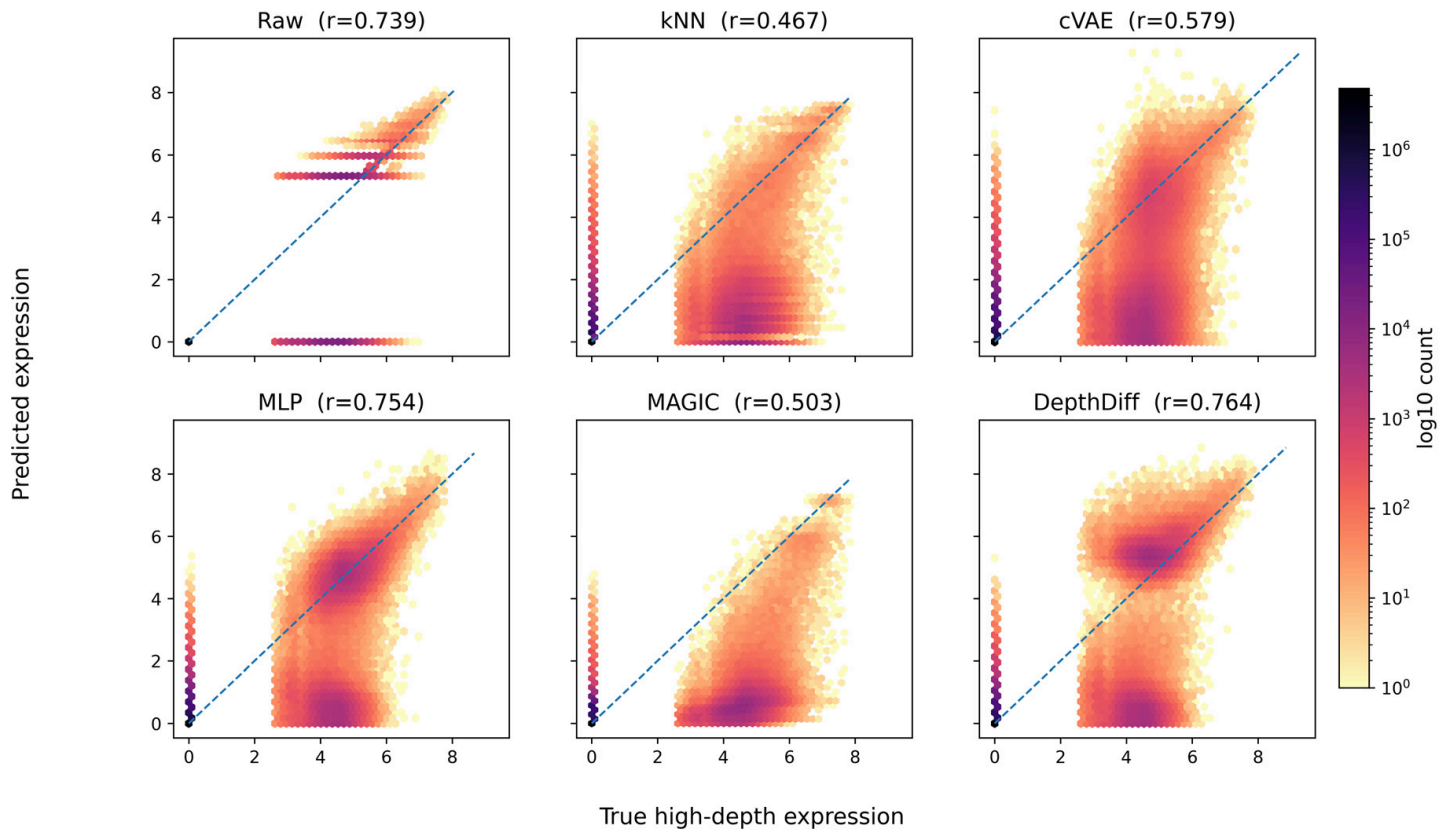
Two-dimensional density plots of predicted versus reference high-depth expression on PBMC68K at 50% sequencing depth. A total of 3000 test cells were randomly sampled for visualization. The panels are arranged as in Figure 3, corresponding to Raw, kNN, cVAE, MLP, MAGIC, and DepthDiff. The global (flat) Pearson correlation for each method is shown in parentheses in the panel title. DepthDiff showed the closest alignment with the diagonal.

**Figure 6:**
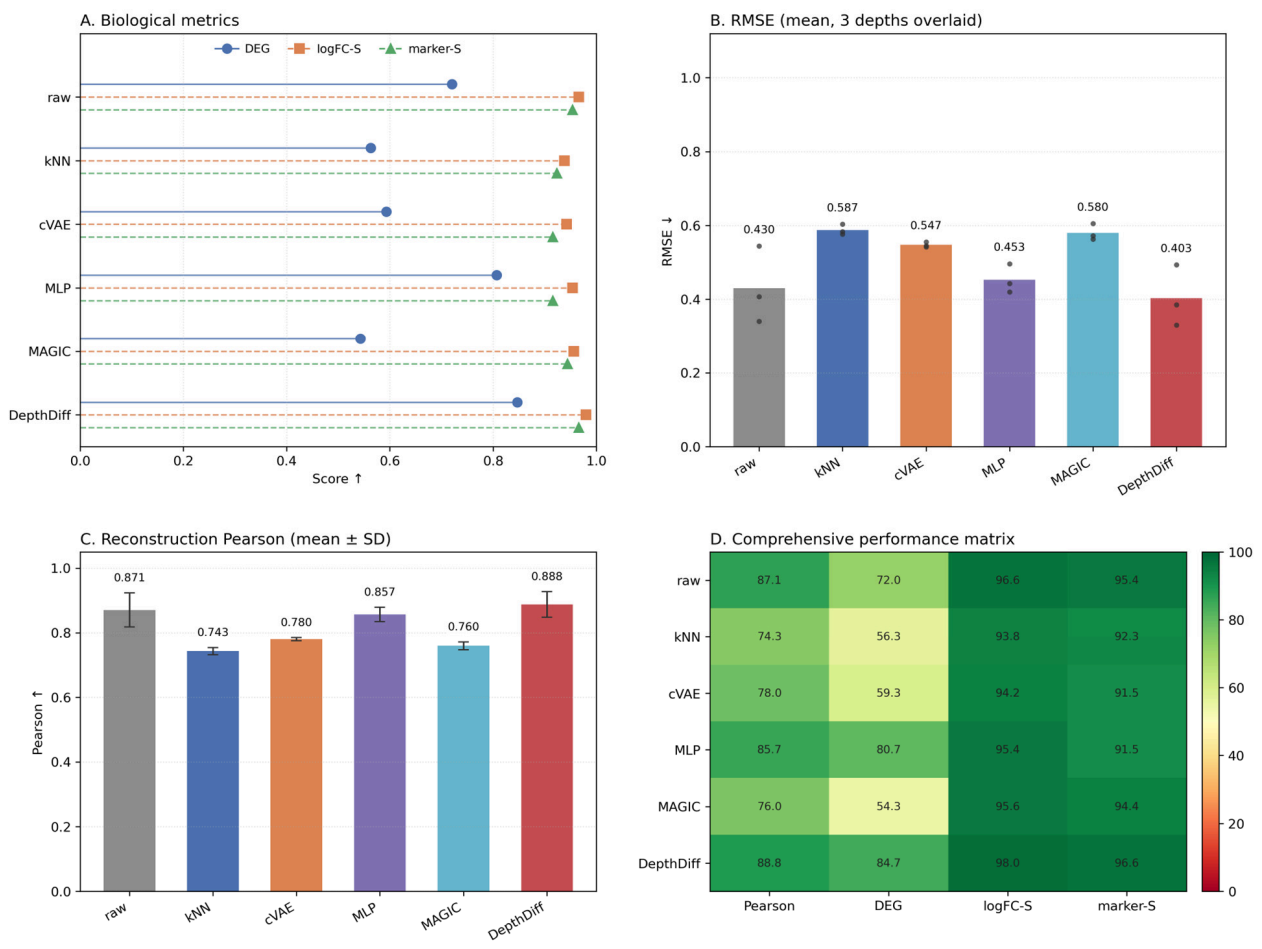
Comprehensive performance of compared methods on the Pancreas dataset. Statistics are averaged across the three sequencing depths for this dataset. The four panels follow the same format as in Figure 2. In this solid-tissue dataset with more complex cell-type composition, DepthDiff achieves the best overall performance across all four panels.

**Figure 7:**
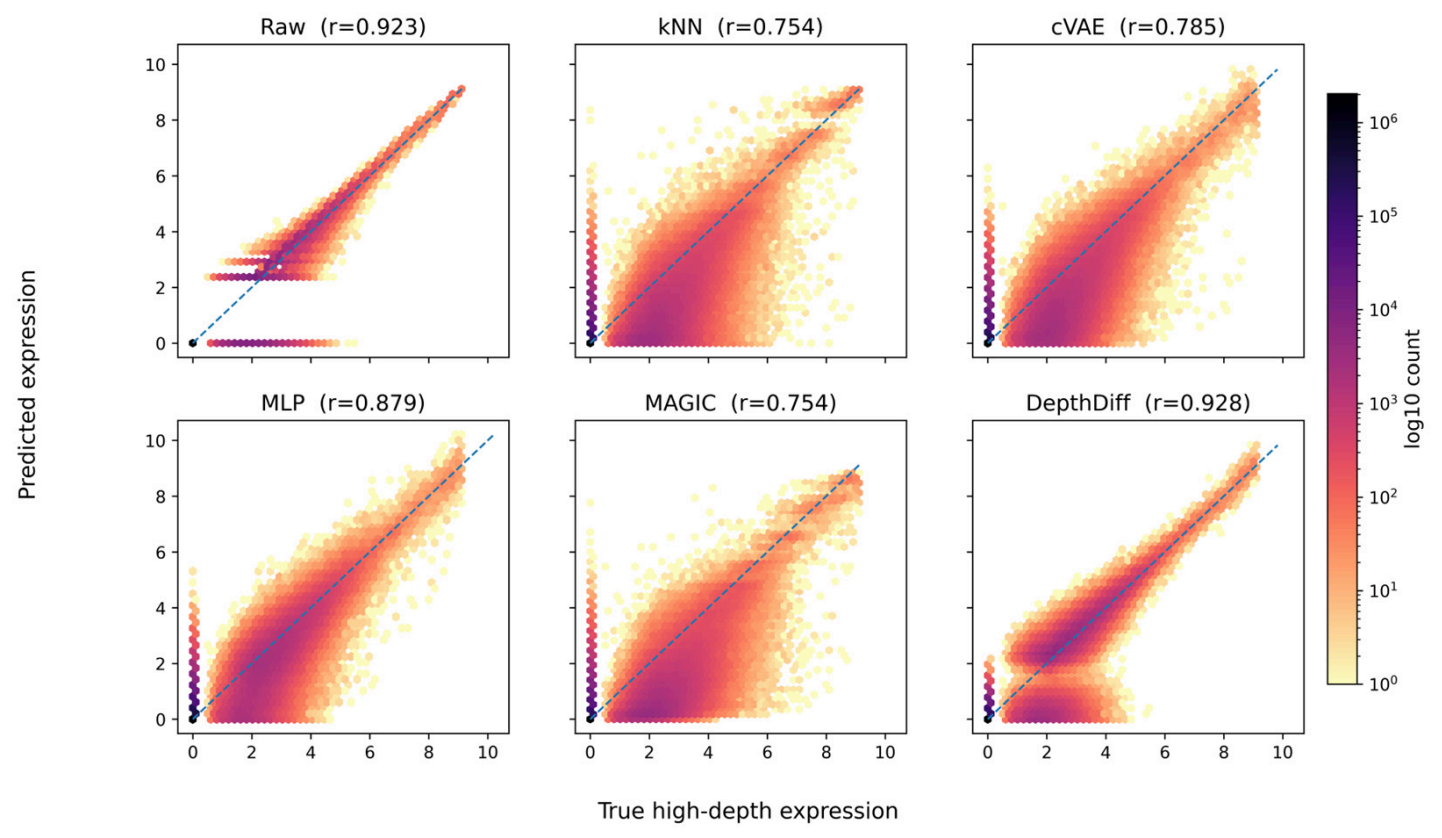
Two-dimensional density plots of predicted versus reference high-depth expression on the Pancreas dataset at 75% sequencing depth. The six panels correspond to Raw, kNN, cVAE, MLP, MAGIC, and DepthDiff. The *x*-axis represents reference high-depth expression, and the *y*-axis represents predicted expression from each method. Color intensity indicates log-scaled count density, and the blue dashed line marks the diagonal. The global (flat) Pearson correlation for each method is shown in parentheses in the panel title. DepthDiff shows the closest alignment with the diagonal.

Table 1 and Table 2 report the averaged performance across all nine conditions. Dataset-specific and depth-specific results, together with composite performance plots and prediction-density plots, are presented in Section 3.2, Section 3.3 and Section 3.4 and Figure 2, Figure 3, Figure 4, Figure 5, Figure 6 and Figure 7. Notably, MAGIC, which was trained only on low-depth data and produced dense smoothed expression values, performed worse than Raw in several reconstruction and differential expression metrics when evaluated against sparse high-depth reference profiles. This observation is consistent with previous single-cell imputation benchmarks and highlights the importance of paired supervision for low-depth-to-high-depth expression enhancement.

### 3.2. Performance on the PBMC3K Dataset

On the widely used PBMC3K benchmark dataset, DepthDiff achieved Pearson correlations of 0.638, 0.780, and 0.872 at 25%, 50%, and 75% sequencing depths, respectively, ranking highest among all compared methods, with a tie with Raw at 75% depth. The corresponding top-100 DEG overlap values were 0.81, 0.86, and 0.87, also ranking best across all depth levels. Notably, the bottleneck-structured supervised latent-variable model cVAE showed markedly weaker DEG recovery performance (0.31–0.43), suggesting a tendency toward over-smoothing. In contrast, DepthDiff outperformed the baselines while maintaining high reconstruction accuracy (Figure 2).

Two-dimensional density plots of predicted versus reference high-depth expression (Figure 3, shown for 25% depth, the sparsest condition) further illustrate these performance differences. Raw low-depth data exhibited clear quantization banding caused by limited sequencing depth; kNN and cVAE showed either value divergence or strong over-smoothing in the low-expression range; MLP displayed systematic expression bias; and MAGIC deviated substantially from the diagonal due to its dense smoothing behavior. By contrast, DepthDiff achieved the closest alignment with the diagonal, with the highest global (flat) Pearson correlation (0.638) among all methods. Statistical significance analysis (Section 3.5) further showed that the per-cell Pearson correlation of DepthDiff was significantly higher than that of all baselines, indicating that its performance advantage was not driven by a small subset of outlier cells.

### 3.3. Robustness Validation on the PBMC68K Dataset

To assess the robustness and scalability of DepthDiff, we further evaluated it on the larger PBMC68K dataset, which contains coarse-grained cluster annotations and exhibits greater intra-cluster heterogeneity. The performance advantage of DepthDiff was consistently replicated and became more pronounced in this large-scale setting. Across the three sequencing depths, DepthDiff achieved Pearson correlations of 0.617, 0.764, and 0.867, exceeding Raw (0.548, 0.739, and 0.858) and the top-performing supervised baseline, MLP (0.606, 0.754, and 0.847). Its top-100 DEG overlap reached 0.70, 0.85, and 0.93, substantially exceeding the corresponding values for Raw and MLP.

These results indicate that, under larger cell numbers and more complex biological signals, DepthDiff maintained superior performance in both per-cell expression reconstruction and differential expression signal preservation, supporting its scalability to large-scale droplet-based scRNA-seq data (Figure 4). Two-dimensional density plots of predicted versus reference high-depth expression (Figure 5, based on 3000 randomly sampled test cells for visualization) further confirmed this pattern. At 50% sequencing depth, DepthDiff achieved the closest alignment with the diagonal and the highest global (flat) Pearson correlation (0.764) among all methods, whereas kNN and cVAE showed clear deviations.

### 3.4. Generalizability Validation on the Pancreas Dataset

On the human pancreas dataset, which has a more complex cell-type composition and represents a solid-tissue scRNA-seq setting beyond PBMC data, DepthDiff achieved the best overall performance. Across the three sequencing depths, it achieved Pearson correlations of 0.834, 0.901, and 0.928, top-100 DEG overlap values of 0.79, 0.84, and 0.91, and the lowest RMSE at all depth levels, outperforming all compared methods (Figure 6).

Two-dimensional density plots of predicted versus reference high-depth expression (Figure 7, Pancreas, 75% sequencing depth) further illustrate these differences. Raw low-depth data exhibits quantization banding caused by limited sequencing depth; kNN and cVAE show clear divergence or over-smoothing in the low-expression range; MLP displays systematic bias; and MAGIC deviates markedly from the diagonal due to its inherent dense smoothing property. By contrast, DepthDiff achieves the closest alignment with the diagonal, with the highest global (flat) Pearson correlation among all methods (r = 0.928). Differential expression fidelity, as measured by logFC Spearman correlation, is also summarized in the composite performance heatmaps for each dataset, where DepthDiff ranks best across all three datasets. Taken together, these results support the generalizability of DepthDiff from immune-cell datasets to solid-tissue scRNA-seq data.

### 3.5. Statistical Significance Analysis

To verify that the performance advantage of DepthDiff is not driven by random fluctuation, we perform paired *t*-tests between DepthDiff and each baseline using per-cell Pearson correlations on the test cells, with the three sequencing depths pooled together. Cohen’s *d* is calculated to quantify effect sizes. Across all datasets and baselines, DepthDiff achieves significantly higher per-cell Pearson correlations, with all comparisons reaching *p* < 1 × 10^−10^.

The effect sizes are moderate relative to Raw (*d* = 0.47–0.76) and MLP (*d* = 0.48–0.70), and large relative to kNN and cVAE (*d* = 1.2–2.1). This pattern is consistent across datasets of different scales, including the smaller PBMC3K dataset (*n* = 1209 cell-depth observations), the larger PBMC68K dataset (*n* = 30,165), and the Pancreas dataset (*n* = 3825). These results support the robustness and generalizability of DepthDiff, rather than a dataset-specific or random effect. Detailed statistics are provided in Table A1 in Appendix A.

### 3.6. Ablation Experiments

To clarify the contribution of each design choice in DepthDiff, we performed ablation experiments on the PBMC3K and Pancreas datasets along three dimensions: prediction parameterization, noise scheduling, and inference strategy (Table 3). All variants used the same network architecture, data splits, number of training epochs, optimizer, and random seed. The reported metrics were averaged across the three sequencing depths.

The ablation results lead to three main observations. First, $x_{0}$-prediction substantially outperformed ϵ-prediction. The ϵ-prediction variants showed sampling divergence on both datasets, particularly under the cosine schedule, because recovering the clean target from predicted noise requires division by $\sqrt{{\bar{\alpha }}_{t}}$, which amplifies noise-prediction errors when ${\bar{\alpha }}_{t}$ is small. Second, cosine scheduling outperformed linear scheduling. The difference was especially large on the Pancreas dataset, where the linear schedule reduced Pearson correlation to 0.520. This is likely because the 50-step linear schedule did not drive the terminal state sufficiently close to pure noise, causing a mismatch between the training endpoint and the inference starting point. Third, full reverse diffusion sampling did not improve performance. Single-step forward prediction matched or slightly exceeded reverse sampling across both datasets, while using 50× fewer network function evaluations (a single forward pass versus 50 reverse steps), corresponding to an approximately 22× wall-clock speedup (Section 3.10).

In addition, the single-step predictor trained with diffusion-based denoising substantially outperformed the direct supervised MLP trained on the same paired data, with Pearson correlations of 0.764 versus 0.639 on PBMC3K and 0.887 versus 0.857 on Pancreas. It should be noted that the difference between DepthDiff and the MLP baseline is not attributable solely to the training objective; it also reflects differences in input construction, as DepthDiff additionally receives the noised residual input $x_{t}$ and the time-step embedding. Therefore, the observed improvement should be interpreted as the contribution of the overall design, namely diffusion-based multi-noise-level denoising training combined with residual and depth conditioning, rather than as the effect of a single isolated component. Nevertheless, because the denoising networks had comparable capacity, with both using residual MLPs with a hidden dimension of 1024, this comparison supports the view that multi-noise-level denoising provides effective regularization and is one of the main contributors to the advantage of DepthDiff over direct supervised regression.

### 3.7. Marker Genes and Biological Interpretation

**Figure 8:**
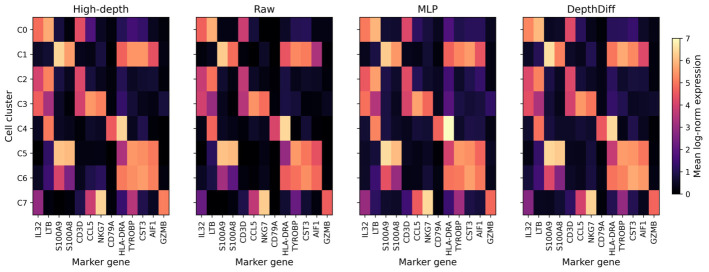
Heatmap of representative canonical marker-gene recovery on PBMC3K at 50% sequencing depth. Rows correspond to KMeans-derived cell clusters, and columns represent representative marker genes for each cluster. The four panels correspond to high-depth, Raw, MLP, and DepthDiff, respectively, with values showing cluster-averaged expression. The expression patterns recovered by DepthDiff are visually most consistent with the high-depth reference.

A key question is how DepthDiff recovers information, particularly for genes whose counts are dropped to zero under low sequencing depth. It is important to clarify that the model does not generate de novo biological signals; rather, it reconstructs the most plausible high-depth-like expression profiles by leveraging high-dimensional transcriptomic dependencies learned from the training data. Figure 8 provides an intuitive illustration of this capability through a heatmap of cluster-averaged expression for representative canonical marker genes in the PBMC3K dataset. Under low-depth conditions (Raw), the cell-type-specific expression patterns of many canonical markers—including S100A8/S100A9 for monocytes, CD3D for T cells, NKG7/GZMB for NK cells, CD79A for B cells, and HLA-DRA/CST3 for myeloid and dendritic cell (DC) populations—are substantially distorted by sampling noise. MLP tends to over-smooth these cell-type-specific signals, whereas DepthDiff more clearly restores the high-expression patterns of these markers in their corresponding cell clusters, yielding expression patterns that are visually most consistent with the high-depth reference.

These findings indicate that DepthDiff preserves key signals that define cell identity at single-cell resolution, thereby improving the reliability of downstream analyses such as cell-type annotation and differential expression analysis.

### 3.8. Cross-Dataset Generalizability Validation

Because strictly paired low- and high-depth expression profiles from independent biological batches are difficult to obtain experimentally, we evaluated whether DepthDiff could generalize across datasets. We trained DepthDiff on one dataset and directly applied it to an independent dataset that was not used during training, involving different donor compositions and sequencing batches. Specifically, we identified 517 shared highly variable genes (HVGs) between PBMC3K and PBMC68K. Using this common gene panel, we trained a cross-dataset model from PBMC68K to PBMC3K and a within-dataset PBMC3K model as an upper-bound baseline trained on the target dataset. Both models were evaluated on the held-out PBMC3K test set (Table 4). Because this analysis was restricted to 517 shared genes, the values were not directly comparable with those in Table 1 and Table 2.

This experiment showed two main findings. First, the low-depth-to-high-depth mapping learned by DepthDiff generalized across independent PBMC datasets. When trained on PBMC68K and applied directly to the unseen PBMC3K test set, the cross-dataset model improved RMSE and Pearson correlation over Raw at all sequencing depths. It also slightly outperformed the within-dataset model on these reconstruction metrics, likely because PBMC68K provided a much larger training set than PBMC3K, approximately 47,000 versus 1900 cells. These results indicate that DepthDiff captures transferable sequencing dropout degradation patterns rather than simply overfitting to the training data.

Second, dataset-specific training remained beneficial for fine-grained differential expression signals. The cross-dataset model produced lower DEG overlap values than the within-dataset model (0.74–0.82 versus 0.83–0.92), likely because DEG recovery depended on the cluster-level structure of PBMC3K, which was not observed during cross-dataset training. Overall, this analysis indicates that DepthDiff generalizes well for expression reconstruction across PBMC datasets, whereas target-dataset-specific training or fine-tuning remains preferable when optimal cluster-level differential expression recovery is required. We further note that DepthDiff operates on a fixed gene panel: its input and output dimensions equal the number of genes in the training feature set, so a source-trained model can only enhance genes present in that panel. Genes that are highly variable in a target dataset but absent from the source panel therefore fall outside the model’s output space and cannot be enhanced by direct transfer; in practice they are handled either by applying the source-trained model on the shared highly variable gene intersection for transferable, reconstruction-level enhancement (as done here with the 517 shared genes), or by training or fine-tuning DepthDiff on the target dataset’s own gene panel—or on the union panel when a combined reference is available—which is also preferable when fine-grained, cluster-level differential expression is the goal.

### 3.9. Orthogonal Validation: Recovery of CITE-Seq Protein-Related Signals

To assess whether the simulated degradation captured the disruption of biological signals caused by low sequencing depth, we performed orthogonal validation using CITE-seq data, which simultaneously measure RNA transcripts and surface proteins in the same cells. Protein expression provides an independent measurement modality that was not used in model training. If the low-depth simulation captures biologically relevant degradation, the concordance between simulated low-depth RNA and protein measurements should decrease. Conversely, if DepthDiff recovers biologically meaningful transcriptomic signals, this RNA–protein concordance should be restored after enhancement.

**Figure 9:**
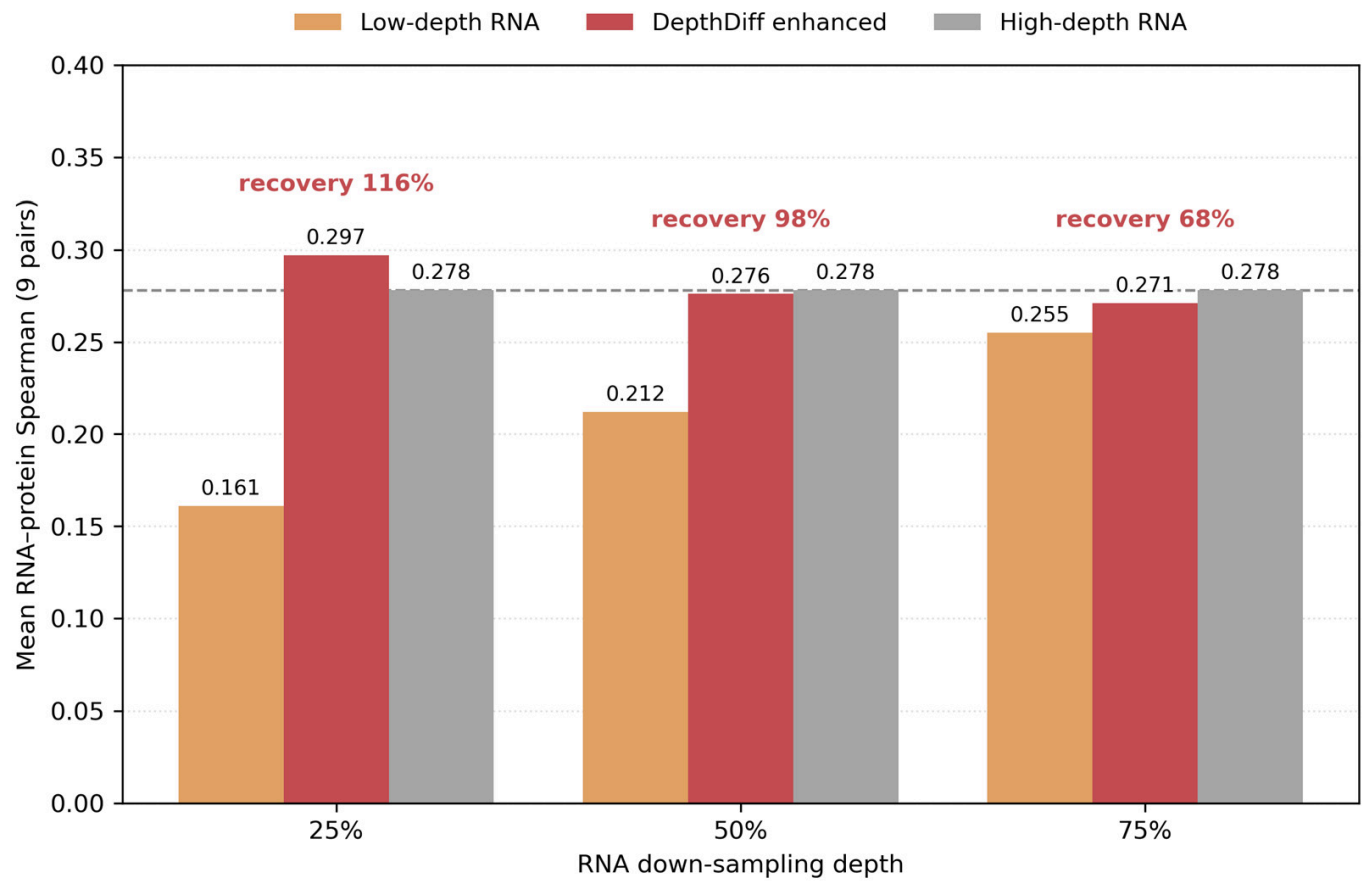
CITE-seq orthogonal validation on the CBMC dataset (GSE100866). The bar plot shows the mean RNA–protein Spearman correlation across nine protein–gene pairs. For each sequencing depth, the three bars correspond to low-depth RNA, DepthDiff-enhanced RNA, and high-depth RNA. The gray dashed line indicates the high-depth reference level (0.278). Values above the bars indicate correlation coefficients, and red annotations indicate recovery rates, calculated as the fraction of lost RNA–protein concordance restored by DepthDiff. Low-depth simulation substantially reduces RNA–protein concordance, whereas DepthDiff enhancement restores it to a level close to or above the high-depth reference.

We used the public CBMC CITE-seq dataset (GEO: GSE100866), which contains 13 antibody-derived tags. After retaining primarily human cells, 8005 cells were included in the analysis. We matched nine reliable protein–gene pairs: CD3–CD3E, CD4–CD4, CD8–CD8A, CD14–CD14, CD16–FCGR3A, CD19–CD19, CD56–NCAM1, CD11c–ITGAX, and CCR7–CCR7. RNA profiles were subjected to fixed-depth downsampling at 25%, 50%, and 75%, followed by enhancement using DepthDiff, while protein measurements were kept unchanged. The results are shown in Figure 9. For each protein–gene pair, RNA–protein Spearman correlation was calculated, and the mean correlation across all pairs was reported. The recovery rate was calculated as the proportion of the low-depth-induced loss in RNA–protein concordance that was restored after enhancement.

The results followed a disruption–recovery pattern. Low-depth simulation reduced the mean RNA–protein concordance from 0.278 under the high-depth reference to 0.161–0.255. After DepthDiff enhancement, the concordance increased to 0.271–0.297, corresponding to recovery rates of 68–116%. At 25% sequencing depth, the enhanced concordance even slightly exceeded the high-depth reference level. This recovery provides important orthogonal evidence that the low-depth simulation introduced structured, biologically relevant technical degradation rather than purely random noise, and that DepthDiff recovered transcriptomic signals consistent with an independent protein modality. It should also be noted that the absolute RNA–surface protein correlations were moderate, which is expected for CITE-seq data because mRNA and protein abundance are not perfectly coupled. Therefore, the key evidence here is the disruption–recovery pattern rather than the absolute correlation magnitude.

### 3.10. Inference Runtime and Computational Efficiency

To provide concrete evidence for the scalability claim, we measured absolute wall-clock inference time on the PBMC68K test set (10,055 cells at 50% sequencing depth) on a single NVIDIA RTX 4060 (Table 5). Single-step DepthDiff enhanced the entire test set in 0.15 s (approximately 65,000 cells/s), which is competitive with the fastest supervised baselines (MLP, 0.10 s; cVAE, 0.11 s) and far faster than the unsupervised MAGIC (20.63 s); MAGIC is transductive and must reprocess the full 67,028-cell matrix to produce test predictions. Relative to the full 50-step reverse-diffusion chain (3.39 s), single-step inference uses 50× fewer network function evaluations and achieves an approximately 22× wall-clock speedup. The gap between the theoretical (50×) and measured (22×) factors reflects fixed data-loading and host–device transfer overheads that are incurred regardless of the number of reverse steps. These results confirm that single-step DepthDiff is well suited to atlas-scale scRNA-seq datasets.

## 4. Discussion

In this study, we proposed DepthDiff, a sequencing-depth-conditioned expression enhancement framework for low-depth single-cell RNA-seq (scRNA-seq) data. Its main contribution lies in integrating conditional diffusion-denoising training with residual learning and explicit depth conditioning, thereby addressing low-depth expression enhancement as a supervised degradation-recovery problem. Across three biologically distinct datasets, DepthDiff achieved strong performance in both numerical expression reconstruction and biological signal preservation, while maintaining efficient single-step inference.

### 4.1. Key Factors: Training Objective Rather than Generative Sampling

Our systematic evaluation provides evidence for the key factors underlying the performance of DepthDiff. First, diffusion-based multi-noise-level denoising training is one of the major contributors to the observed improvement. The ablation experiments showed that the single-step predictor trained with the same paired data and comparable network capacity substantially outperformed direct MLP regression, for example with Pearson correlations of 0.764 versus 0.639 on PBMC3K. Multi-noise-level denoising can be viewed as a form of data augmentation and regularization, forcing the model to learn stable low-depth-to-high-depth mappings under different degradation levels, which may improve generalization compared with a single MSE-based regression objective.

It should be noted, however, that this comparison does not completely disentangle the training objective from the input construction, because DepthDiff additionally incorporates the noised residual input and time-step embedding. Therefore, the performance gain should be interpreted as arising from the overall design, namely diffusion-based denoising training combined with residual and depth-conditioned modeling, rather than from a single isolated component. Future ablations with controlled input structures would help further separate the individual contributions of these factors.

Second, $x_{0}$-prediction and cosine noise scheduling are important for training and inference stability. The ϵ-prediction variants diverged under the cosine schedule because recovering the clean target from predicted noise requires division by $\sqrt{{\bar{\alpha }}_{t}}$, which amplifies noise-prediction errors when ${\bar{\alpha }}_{t}$ is small. In contrast, $x_{0}$-prediction avoids this instability. Linear scheduling also performed worse because the terminal state after 50 noise levels was not sufficiently close to pure noise, resulting in a mismatch between the endpoint of the forward process and the starting point used during inference.

Third, full reverse diffusion sampling is not necessary for this task. The ablation results showed that single-step prediction matched or slightly outperformed multi-step reverse sampling across the evaluated metrics, including gene-variance preservation, while using 50× fewer network function evaluations and achieving an approximately 22× wall-clock speedup (Section 3.10, Table 5). This finding has both methodological and practical implications: for low-depth scRNA-seq enhancement, the main benefit comes from diffusion-based denoising training rather than generative reverse sampling, and the single-step design makes the method more suitable for large-scale omics applications.

It is also notable that the latent-variable-based supervised model cVAE performed worse than both direct MLP regression and DepthDiff. This suggests that the advantage of DepthDiff does not simply arise from more complex latent-space compression, but from the combination of denoising training, residual modeling, and sequencing-depth conditioning.

### 4.2. Depth Enhancement and Unsupervised Imputation Represent Different Problem Settings

Our comparison highlights an important distinction that is often overlooked: low-depth expression enhancement and unsupervised imputation are related but fundamentally different problem settings. Unsupervised imputation methods, such as MAGIC [4], scVI [6], DCA [5], and ALRA [25], aim to recover dropout-related signals within the observed low-depth data distribution. Their objectives are based on count likelihood, latent-variable reconstruction, local smoothing, or low-rank approximation, and they do not have access to a paired high-depth target distribution.

By contrast, the depth-enhancement problem studied here is a supervised mapping-learning task. It uses paired high-depth expression profiles as reference labels and directly learns the degradation inverse from low-depth to high-depth-like expression. Because the two problem settings optimize different objectives, their appropriate use cases also differ. When sparse high-depth profiles are used as the reference and the evaluation focuses on reconstruction accuracy and differential expression fidelity, unsupervised imputation methods can underperform paired supervised enhancement methods. In our benchmark, MAGIC performed worse than Raw in Pearson correlation and DEG overlap. This result does not imply that unsupervised imputation methods are generally ineffective; rather, they address a different problem.

This distinction also explains the source of DepthDiff’s improvement. By explicitly modeling low sequencing depth as a depth-conditioned, learnable degradation process and supervising its inverse with paired data, DepthDiff is more directly aligned with the goal of approximating high-depth reference expression than methods that smooth within the observed distribution. The two approaches should therefore be regarded as complementary. In purely exploratory settings where no high-depth reference is available and paired simulation is not feasible, unsupervised imputation remains a useful option. When high-quality references are available, or when fixed-UMI paired samples can be constructed from them, paired supervised enhancement can provide more consistent gains. Thus, the choice of method should depend on whether usable high-depth supervision is available, rather than on a general ranking between supervised enhancement and unsupervised imputation.

### 4.3. Practical Guidance for Downstream Analysis

Based on the results, we provide several practical recommendations for users. First, DepthDiff is most suitable for expression reconstruction, differential expression analysis, logFC estimation, and marker gene analysis. It consistently achieved the strongest performance in these metrics across three datasets and three sequencing depths, with particularly clear gains at 25% sequencing depth, where degradation was most severe.

Second, clustering-based analyses should be treated with caution. DepthDiff was not always optimal for clustering metrics such as ARI and NMI, and expression enhancement may alter local distance structures. For analyses centered on clustering, we recommend evaluating both raw and enhanced data before deciding whether enhancement should be used.

Third, the learned low-depth-to-high-depth mapping can be transferred across independent datasets, as shown in Section 3.8. A model trained on a large reference dataset, such as PBMC68K, can be directly applied to a new dataset for reconstruction-level enhancement. However, when the goal is optimal fine-grained cluster-level differential expression recovery, training or fine-tuning with paired data from the target dataset remains preferable.

Fourth, the single-step inference design improves computational efficiency. Because DepthDiff does not require iterative reverse sampling, it is more scalable to atlas-level scRNA-seq datasets than conventional multi-step diffusion sampling procedures.

Finally, the enhanced nonzero values should be interpreted as denoised estimates inferred from learned gene–gene dependencies, rather than as newly measured observations. Key biological conclusions should still be cross-validated using raw data or independent evidence whenever possible, particularly when interpreting signals that are restored by the model.

### 4.4. Applications and Limitations

High-fidelity enhancement of low-depth scRNA-seq data may enable more reliable use of technically compromised or low-depth datasets in biological studies. Potential applications include large-scale atlas construction and cost-constrained clinical screening, where improved marker gene detection, differential expression analysis, and pathway-level interpretation may be valuable.

This study has several limitations. First, the benchmarks were based on fixed-UMI low-depth simulations generated from high-depth data. This design provides controllability and paired supervision, but it cannot fully capture all sources of low-quality scRNA-seq data in real experiments, such as library preparation failure, batch effects, platform differences, ambient RNA [26], or degraded samples. In particular, whereas our fixed-UMI simulation already applies gene-specific capture variation and a low-expression-dependent dropout tendency, real low-depth data may exhibit more strongly non-uniform, gene- and platform-dependent dropout, together with ambient-RNA contamination, amplification bias, inefficient RNA capture, and low library complexity; such confounders can introduce a distribution shift between simulated and genuine biological low-depth samples that could reduce the effectiveness of a model trained solely on simulated pairs. Moreover, several of these confounders corrupt the data in qualitatively different ways from sequencing dropout—ambient RNA and doublets add spurious counts rather than removing them, and RNA degradation produces position-dependent coverage loss—whereas DepthDiff is trained to invert a count-removing thinning process; its residual, depth-conditioned formulation is therefore best suited to depth-driven dropout, and additive or coverage-biased corruptions may require an explicitly extended degradation model. Although the cross-dataset transfer analysis and CITE-seq orthogonal validation provide indirect evidence that DepthDiff captures transferable, biologically relevant degradation rather than simulation-specific artifacts, its generalization to experimentally generated low-depth data has not yet been directly established; calibrating the degradation model against, and validating DepthDiff on, real low-quality samples is therefore an important direction for future work. Second, although our comparison included supervised baselines and the unsupervised method MAGIC, additional imputation methods such as scVI, DCA, ALRA, and SAVER [27,28] were not systematically included in the main benchmark. Many of these methods are unsupervised and optimize objectives that are not fully aligned with sparse high-depth reconstruction, but broader benchmarking would still be informative. Third, DepthDiff was not always optimal for clustering metrics such as ARI and NMI, suggesting that expression enhancement may alter local neighborhood structures and should be applied cautiously in clustering-centered workflows.

Fourth, the current denoising network was implemented as a residual MLP. More complex architectures, such as Transformer-based models [21], Mamba-style sequence models [29], or models incorporating gene-network priors [30,31], were not systematically evaluated. Fifth, although cross-dataset transfer analysis and CITE-seq protein-based orthogonal validation supported the generalizability and biological relevance of DepthDiff, the present study did not include validation on experimentally generated low-depth or low-quality samples, such as truly shallow-sequenced or FFPE-derived scRNA-seq data. Obtaining real paired samples and evaluating DepthDiff in such settings will be an important direction for future work. Sixth, our benchmarks were confined to droplet-based UMI platforms (10x Genomics and inDrop). In its formulation, DepthDiff is platform-agnostic and operates on the normalized highly variable gene expression matrix, and we therefore expect it to transfer most directly to other droplet-based, UMI-tagged 3′ protocols (e.g., Drop-seq, CEL-seq2), which share the molecule-count statistics and dropout behavior that our depth-conditioning and fixed-UMI simulation assume. Extending it to plate-based, full-length protocols such as Smart-seq2 is less straightforward: these lack UMIs, quantify reads rather than molecules, and exhibit length- and coverage-dependent biases, so both the degradation model used to construct paired training data and the normalization would need to be adapted to the platform’s noise structure. We therefore regard cross-platform extension, particularly to non-UMI protocols, as a concrete and worthwhile direction for future work.

## 5. Conclusions

In this study, we proposed DepthDiff, a sequencing-depth-conditioned expression enhancement model trained with a diffusion-denoising objective. DepthDiff combines residual diffusion targets, $x_{0}$-prediction, and cosine noise scheduling to handle multiple sequencing depths within a single model, while enabling efficient inference through single-step forward prediction. Systematic evaluation across three datasets and three sequencing depths showed that single-step DepthDiff consistently outperformed supervised baselines, the unsupervised method MAGIC, and unenhanced low-depth data in expression reconstruction and downstream biological signal preservation, including differential expression analysis. Ablation experiments further indicated that the performance gains mainly arose from diffusion-based denoising training and the $x_{0}$-prediction/cosine-scheduling design, rather than from generative reverse sampling.

DepthDiff provides a reproducible, scalable, and computationally efficient framework for recovering high-depth-like expression profiles and biologically relevant signals from low-depth scRNA-seq data. Its contribution does not lie in proposing a new generative diffusion model [32,33], but in demonstrating that task-specific integration of multi-noise-level diffusion-denoising training, residual supervised regression, and sequencing-depth conditioning can be effectively implemented with single-step inference for low-depth single-cell expression enhancement.

## Figures and Tables

**Table 1:** Technical metrics averaged across three datasets and three sequencing depths.

Method	RMSE	Pearson
Raw	0.600	0.776
kNN	0.777	0.583
cVAE	0.737	0.636
MLP	0.638	0.744
MAGIC	0.781	0.610
DepthDiff	0.570	0.800

**Table 2:** Biological signal metrics averaged across three datasets and three sequencing depths.

Method	DEG Overlap	LogFC-S	Marker-S
Raw	0.712	0.762	0.796
kNN	0.580	0.748	0.677
cVAE	0.501	0.781	0.758
MLP	0.764	0.787	0.771
MAGIC	0.322	0.694	0.740
DepthDiff	0.840	0.889	0.862

**Table 3:** Ablation analysis of key DepthDiff design choices.

Parameterization	Schedule	Inference	PBMC3K	Pancreas
$x_{0}$	cosine	single-step (default)	0.764/0.85	0.887/0.85
$x_{0}$	cosine	reverse sampling	0.740/0.84	0.876/0.84
ϵ	cosine	reverse sampling	divergent/0.05	divergent/0.08
$x_{0}$	linear	reverse sampling	0.711/0.81	0.520/0.77
ϵ	linear	reverse sampling	0.681/0.66	divergent/0.23

**Table 4:** Cross-dataset generalizability analysis on the PBMC3K test set using 517 shared highly variable genes.

Depth	Method	RMSE	Pearson	DEG Overlap	logFC-S
25%	Raw	1.052	0.620	0.82	0.858
	DepthDiff, within-dataset (3K → 3K)	0.939	0.689	0.83	0.925
	DepthDiff, cross-dataset (68K → 3K)	0.901	0.713	0.74	0.898
50%	Raw	0.806	0.790	0.85	0.920
	DepthDiff, within-dataset (3K → 3K)	0.757	0.811	0.88	0.949
	DepthDiff, cross-dataset (68K → 3K)	0.729	0.823	0.79	0.942
75%	Raw	0.610	0.883	0.89	0.948
	DepthDiff, within-dataset (3K → 3K)	0.591	0.889	0.92	0.963
	DepthDiff, cross-dataset (68K → 3K)	0.571	0.895	0.82	0.966

**Table 5:** Inference runtime on the PBMC68K test set (10,055 cells, 50% sequencing depth; single NVIDIA RTX 4060; neural methods on GPU, MAGIC on CPU).

Method	Runtime (s)
MLP	0.10
cVAE	0.11
DepthDiff (single-step)	0.15
DepthDiff (full reverse, 50 steps)	3.39
MAGIC	20.63

## Data Availability

The source code developed for this study (DepthDiff, version 1.0.0) is openly available on GitHub at https://github.com/yunduomenghuale/DepthDiff (accessed on 21 June 2026). All datasets analyzed in this study are publicly available from the following sources: the PBMC3K dataset (https://www.10xgenomics.com/datasets/3-k-pbm-cs-from-a-healthy-donor-1-standard-1-1-0, accessed on 8 April 2026); the PBMC68K dataset (Fresh 68k PBMCs, Donor A; https://www.10xgenomics.com/datasets/fresh-68-k-pbm-cs-donor-a-1-standard-1-1-0, accessed on 22 April 2026); the human pancreas scRNA-seq dataset (GEO: GSE84133, https://www.ncbi.nlm.nih.gov/geo/query/acc.cgi?acc=GSE84133, accessed on 12 May 2026); and the CBMC CITE-seq dataset (GEO: GSE100866, https://www.ncbi.nlm.nih.gov/geo/query/acc.cgi?acc=GSE100866, accessed on 28 May 2026).

## Abbreviations

The following abbreviations are used in this manuscript:

ALRA	Adaptively thresholded Low-Rank Approximation
ARI	Adjusted Rand Index
CBMC	Cord Blood Mononuclear Cell
CITE-seq	Cellular Indexing of Transcriptomes and Epitopes by Sequencing
cVAE	Conditional Variational Autoencoder
DCA	Deep Count Autoencoder
DDIM	Denoising Diffusion Implicit Model
DDPM	Denoising Diffusion Probabilistic Model
DEG	Differentially Expressed Gene
HVG	Highly Variable Gene
kNN	k-Nearest Neighbors
logFC	Log Fold Change
MAGIC	Markov Affinity-based Graph Imputation of Cells
MLP	Multilayer Perceptron
MSE	Mean Squared Error
NMI	Normalized Mutual Information
PBMC	Peripheral Blood Mononuclear Cell
RMSE	Root Mean Square Error
scVI	single-cell Variational Inference
scRNA-seq	Single-cell RNA Sequencing
UMI	Unique Molecular Identifier

## Appendix A

**Table A1:** Statistical significance analysis based on paired *t*-tests of per-cell Pearson correlations between DepthDiff and each baseline, with the three sequencing depths pooled together.

Dataset	Comparison	Sample Size	DepthDiff Mean	Baseline Mean	*p*-Value	Cohen’s *d*
PBMC3K	vs. Raw	1209	0.769	0.747	<1 × 10^−10^	0.470
	vs. kNN	1209	0.769	0.532	<1 × 10^−10^	1.381
	vs. cVAE	1209	0.769	0.552	<1 × 10^−10^	1.246
	vs. MLP	1209	0.769	0.641	<1 × 10^−10^	1.203
PBMC68K	vs. Raw	30,165	0.759	0.727	<1 × 10^−10^	0.660
	vs. MLP	30,165	0.759	0.743	<1 × 10^−10^	0.704
	vs. kNN/cVAE	30,165	0.759	0.48–0.57	<1 × 10^−10^	1.77–2.07
Pancreas	vs. Raw	3825	0.880	0.860	<1 × 10^−10^	0.762
	vs. MLP	3825	0.880	0.859	<1 × 10^−10^	0.478
	vs. kNN/cVAE	3825	0.880	0.74–0.78	<1 × 10^−10^	1.11–1.21
